# A Case of Pancreatic Cancer Complicated by Active Pulmonary Tuberculosis Successfully Managed through a Multidisciplinary Treatment Strategy

**DOI:** 10.70352/scrj.cr.26-0532

**Published:** 2026-10-02

**Authors:** Kenta Tanaka, Masaya Suenaga, Mitsuru Tashiro, Takuma Umemura, Takumi Hada, Hiroshi Hakamata, Hisako Tajima, Yasuo Uno, Masashi Hattori, Yasuhiro Kodera

**Affiliations:** Department of Surgery, NHO Nagoya Medical Center, Nagoya, Aichi, Japan

**Keywords:** pancreatic cancer, pancreatic ductal adenocarcinoma, pulmonary tuberculosis, antituberculosis therapy, neoadjuvant chemotherapy, pancreatoduodenectomy

## Abstract

**INTRODUCTION:**

Pancreatic cancer complicated by active pulmonary tuberculosis (TB) presents a therapeutic dilemma because timely oncologic treatment must be balanced against infection control. Evidence guiding the curative-intent management of pancreatic cancer complicated by active TB remains extremely limited.

**CASE PRESENTATION:**

An 81-year-old man with a history of tuberculous peritonitis and diabetes mellitus presented with weight loss and poor glycemic control. Contrast-enhanced CT revealed a 14-mm pancreatic head tumor and multiple nodular lesions in the right upper lobe. Sputum examination was positive for acid-fast bacilli and *Mycobacterium tuberculosis*. After 4 weeks of initial 4-drug anti-TB therapy, sputum smear conversion and drug-susceptible TB were confirmed. Re-evaluation imaging revealed enlargement of the tumor to 21 mm with less than 180° portal vein abutment, and endoscopic US-guided fine-needle aspiration confirmed pancreatic ductal adenocarcinoma (PDAC). Although the tumor was anatomically resectable, active pulmonary TB was considered a host-related risk, and treatment was planned according to a strategy analogous to the International Association of Pancreatology concept of borderline resection on the basis of conditional factors. Following multidisciplinary discussion and shared decision-making with the patient, concurrent maintenance anti-TB therapy and 6 cycles of neoadjuvant gemcitabine plus nab-paclitaxel (GnP) were administered. The planned treatment was completed successfully without TB reactivation, resulting in a partial radiological response that permitted subtotal stomach-preserving pancreatoduodenectomy with portal vein resection. Histopathological examination revealed an R0 resection with a Grade 1b therapeutic response. After completing adjuvant chemotherapy, the patient developed a local recurrence 16 months after surgery but remained alive with stable disease under systemic chemotherapy 33 months after the initiation of anti-TB therapy, without TB reactivation.

**CONCLUSIONS:**

Although further accumulation of similar cases is needed, this case demonstrates that curative-intent treatment of PDAC complicated by active TB can be achieved through careful multidisciplinary management.

## Abbreviations

BR-C
: borderline resectable based on conditional criteria
CA19-9
: carbohydrate antigen 19-9
CYP
: cytochrome P450
EB
: ethambutol
GnP
: gemcitabine plus nab-paclitaxel
IAP
: International Association of Pancreatology
INH
: isoniazid
MGIT
: mycobacterial growth indicator tube
PDAC
: pancreatic ductal adenocarcinoma
PZA
: pyrazinamide
RECIST
: Response Evaluation Criteria in Solid Tumors
RFP
: rifampicin
S-1
: tegafur/gimeracil/oteracil
Span-1
: s-pancreas antigen-1
TB
: tuberculosis

## INTRODUCTION

TB remains a major global health problem and continues to be one of the leading causes of death from a single infectious agent worldwide.^1)^ Although the incidence of primary TB has declined in many developed countries, the reactivation of latent infection remains an important clinical issue. In this context, malignancy is a recognized risk factor for TB reactivation, together with diabetes mellitus and other immunocompromised conditions, owing to cancer-associated malnutrition, immune dysregulation, and the effects of anticancer therapies.^2)^

Concomitant TB and malignancy have been reported most frequently in patients with lung cancer and hematologic malignancies, whereas coexistence with pancreatic cancer, one of the most lethal solid malignancies, is relatively rare.^2,3)^ Furthermore, reports describing patients who require concurrent anti-TB therapy and active cancer treatment remain limited.^4)^ Consequently, the management of these patients is often challenging because clinicians must balance infection control and the prevention of TB reactivation against timely oncologic treatment. We report a rare case of pancreatic cancer complicated by active pulmonary TB that was successfully managed through a multidisciplinary treatment strategy combining anti-TB therapy and neoadjuvant chemotherapy before curative-intent surgical resection.

## CASE PRESENTATION

An 81-year-old man was referred to our hospital for further evaluation of a 10-kg weight loss over the preceding 6 months and worsening glycemic control. His medical history included hypertension, diabetes mellitus, and a remote history of tuberculous peritonitis at 19 years of age. On physical examination, the abdomen was flat and soft, and the Eastern Cooperative Oncology Group performance status was 1. Laboratory tests revealed hyperglycemia (glucose, 178 mg/dL; hemoglobin A1c, 12.5%) and elevated tumor marker levels, including a CA19-9 concentration of 370 U/mL and an Span-1 concentration of 63 U/mL. No other significant abnormalities were identified. Contrast-enhanced CT revealed a 14-mm hypoenhancing mass in the pancreatic head with dilatation of the main pancreatic duct (**Fig. 1A** and **1B**). No regional lymph node metastasis or distant metastasis was evident on CT. However, multiple calcified intra-abdominal lymph nodes, presumably related to the patient’s previous tuberculous peritonitis, were noted (**Fig. 1C**). In addition, multiple nodular lesions were identified in the right upper lobe of the lung (**Fig. 1D**).

**Fig. 1:**
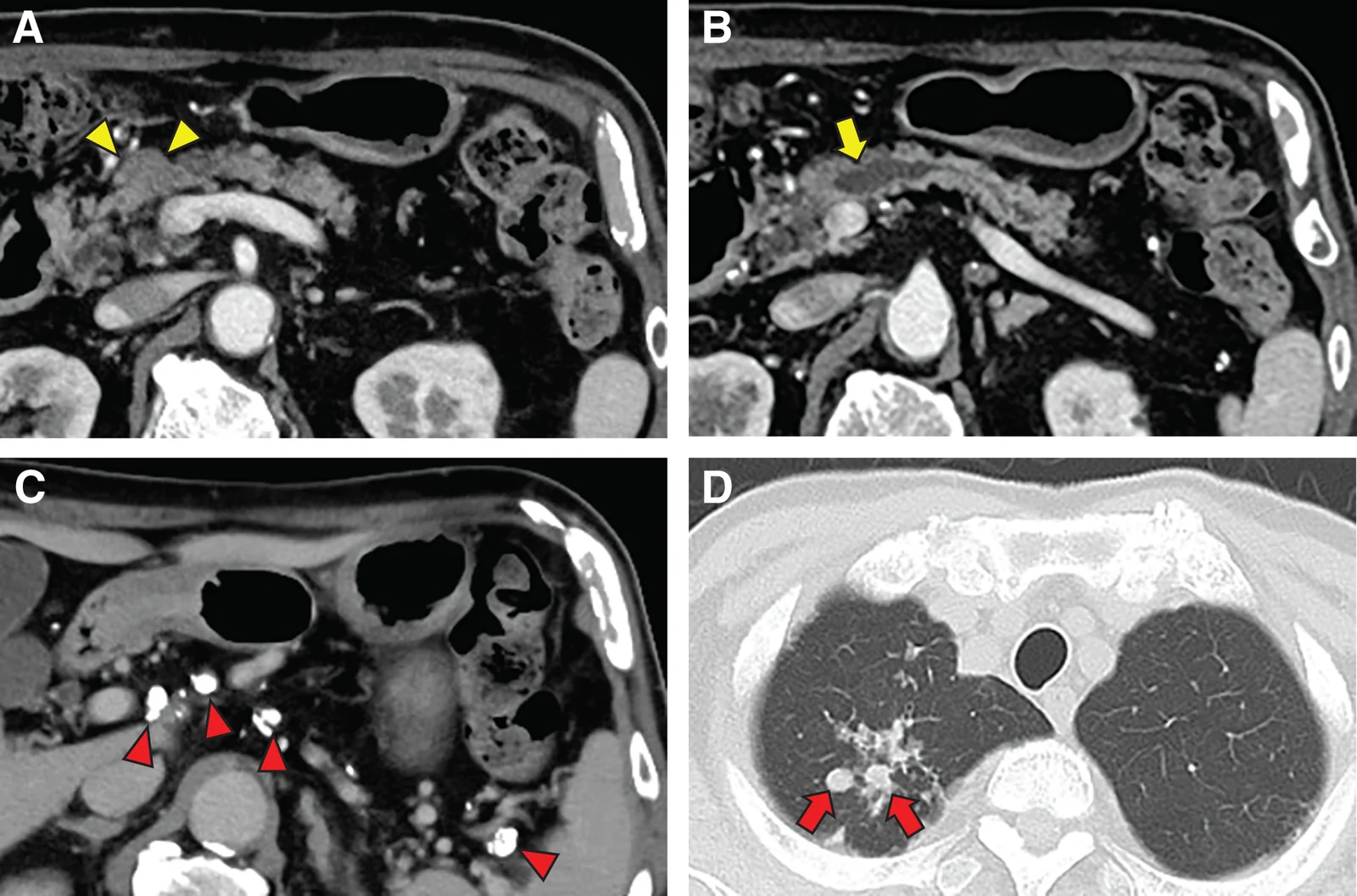
Contrast-enhanced CT findings before treatment. (**A**) A 14-mm hypoenhancing mass was observed in the pancreatic head (yellow arrowhead). (**B**) The main pancreatic duct was dilated (yellow arrow). (**C**) Multiple calcified lymph nodes were identified in the abdomen (red arrowhead). (**D**) Multiple nodular opacities were observed in the right upper lobe of the lung (red arrow).

Urgent evaluation of the pulmonary lesions revealed acid-fast bacilli on sputum smears and *Mycobacterium tuberculosis* DNA by polymerase chain reaction, leading to a diagnosis of active pulmonary TB. Because active TB with ongoing bacterial shedding poses an infection control concern, anti-TB therapy was initiated before oncologic assessment. In accordance with Japanese infectious disease regulations, the patient was transferred to a specialized TB isolation ward and received initial anti-TB therapy with RFP, INH, PZA, and EB, which is consistent with the standard initial regimen for presumed drug-susceptible pulmonary TB.^1)^ After 4 weeks of anti-TB therapy, 3 consecutive sputum smear examinations were negative, confirming sputum smear conversion. In addition, drug susceptibility testing using the MGIT method demonstrated susceptibility to all tested anti-TB agents, and molecular testing detected no RFP resistance mutations. The patient was discharged from the TB ward and returned to our hospital for further diagnostic and oncologic evaluation while continuing anti-TB therapy.

A re-evaluation CT showed enlargement of the pancreatic tumor to 21 mm with less than 180° portal vein abutment (**Fig. 2A** and **2B**). The tumor marker levels remained stable during anti-TB therapy (**Fig. 3**). Endoscopic US-guided fine-needle aspiration was subsequently performed, and pathological examination confirmed PDAC. In accordance with the IAP consensus criteria,^5)^ the tumor was anatomically resectable. Moreover, after completing the initial 2-month, 4-drug regimen, TB specialists recommended continuation of 2-drug maintenance anti-TB therapy for a total treatment duration of 9 months because of the patient’s diabetes mellitus and underlying malignancy, in accordance with Japanese TB treatment guidelines.^6)^ Considering the need for continued anti-TB therapy to minimize the risk of TB reactivation, as well as the possibility that TB reactivation or further deterioration in performance status could render the tumor unresectable, the case was discussed at a multidisciplinary conference involving surgeons, pulmonologists, and anesthesiologists. Although the patient did not strictly meet the IAP definition of borderline resectable based on the conditional criteria (BR-C), treatment was planned in a manner analogous to the BR-C concept. Given the rarity of concomitant PDAC and active TB and the limited evidence regarding the safety and efficacy of concurrent anti-TB therapy and neoadjuvant chemotherapy, the proposed treatment strategy was carefully discussed with the patient, and informed consent was obtained. Concurrent neoadjuvant GnP and maintenance anti-TB therapy were therefore selected, with 6 cycles of GnP scheduled to coincide with the remaining course of the 9-month anti-TB therapy before surgery. Given the patient’s advanced age, GnP was initiated at doses of gemcitabine 820 mg/m^2^ and nab-paclitaxel 105 mg/m^2^ on days 1, 8, and 15 every 4 weeks while maintaining the planned treatment schedule.^7)^ Dexamethasone 6.6 mg was administered intravenously as antiemetic premedication on each GnP treatment day, for a total of 18 intermittent doses over 6 cycles.

**Fig. 2:**
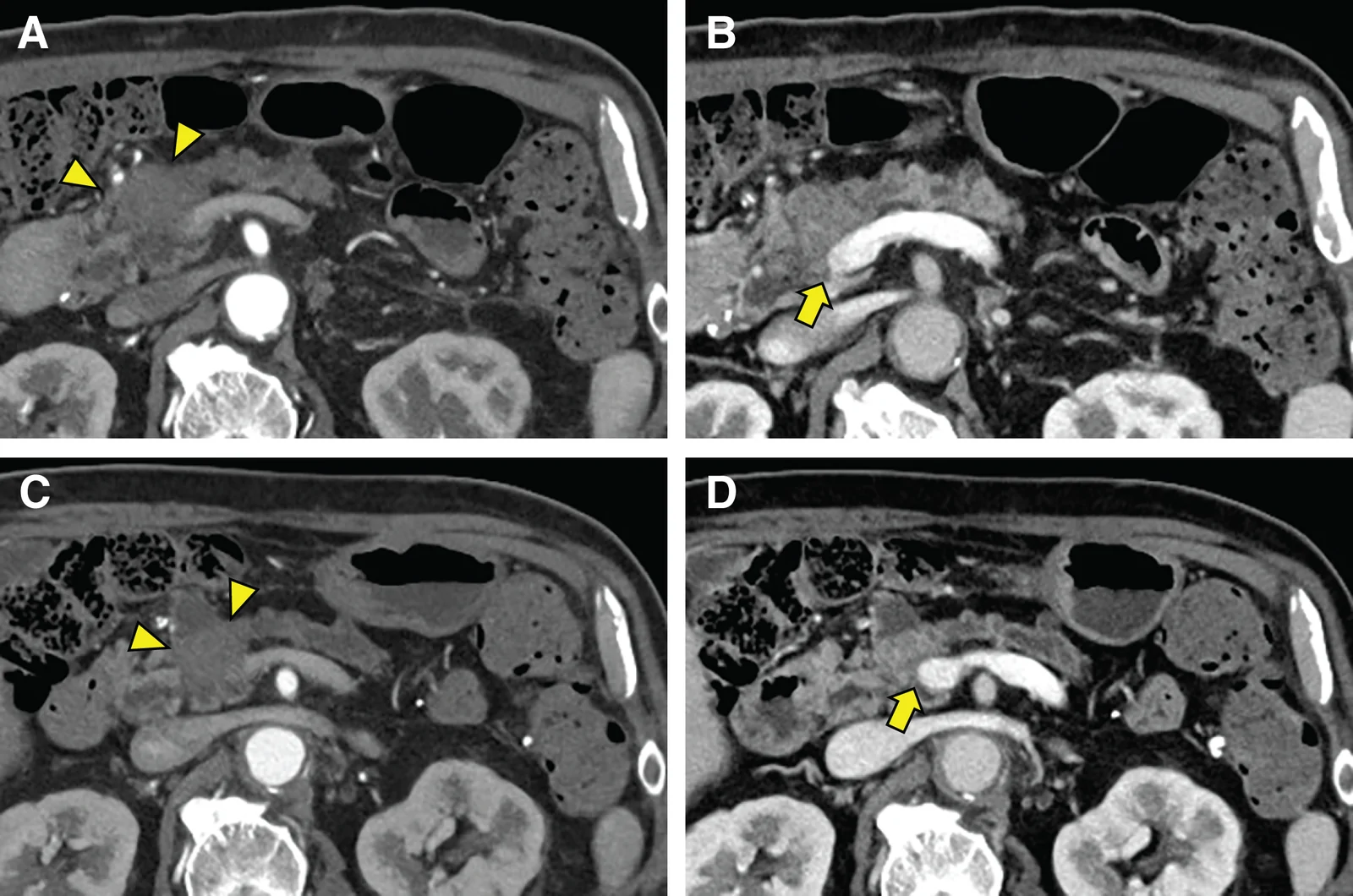
Contrast-enhanced CT findings before and after neoadjuvant chemotherapy. (**A**, **B**) Before neoadjuvant chemotherapy, the tumor enlarged to 21 mm (yellow arrowhead) and showed less than 180° portal vein abutment (yellow arrow). (**C**, **D**) After neoadjuvant chemotherapy, the tumor shrank to 14 mm (yellow arrowhead), representing a 33% reduction, while the degree of portal vein abutment remained unchanged (yellow arrow).

**Fig. 3:**
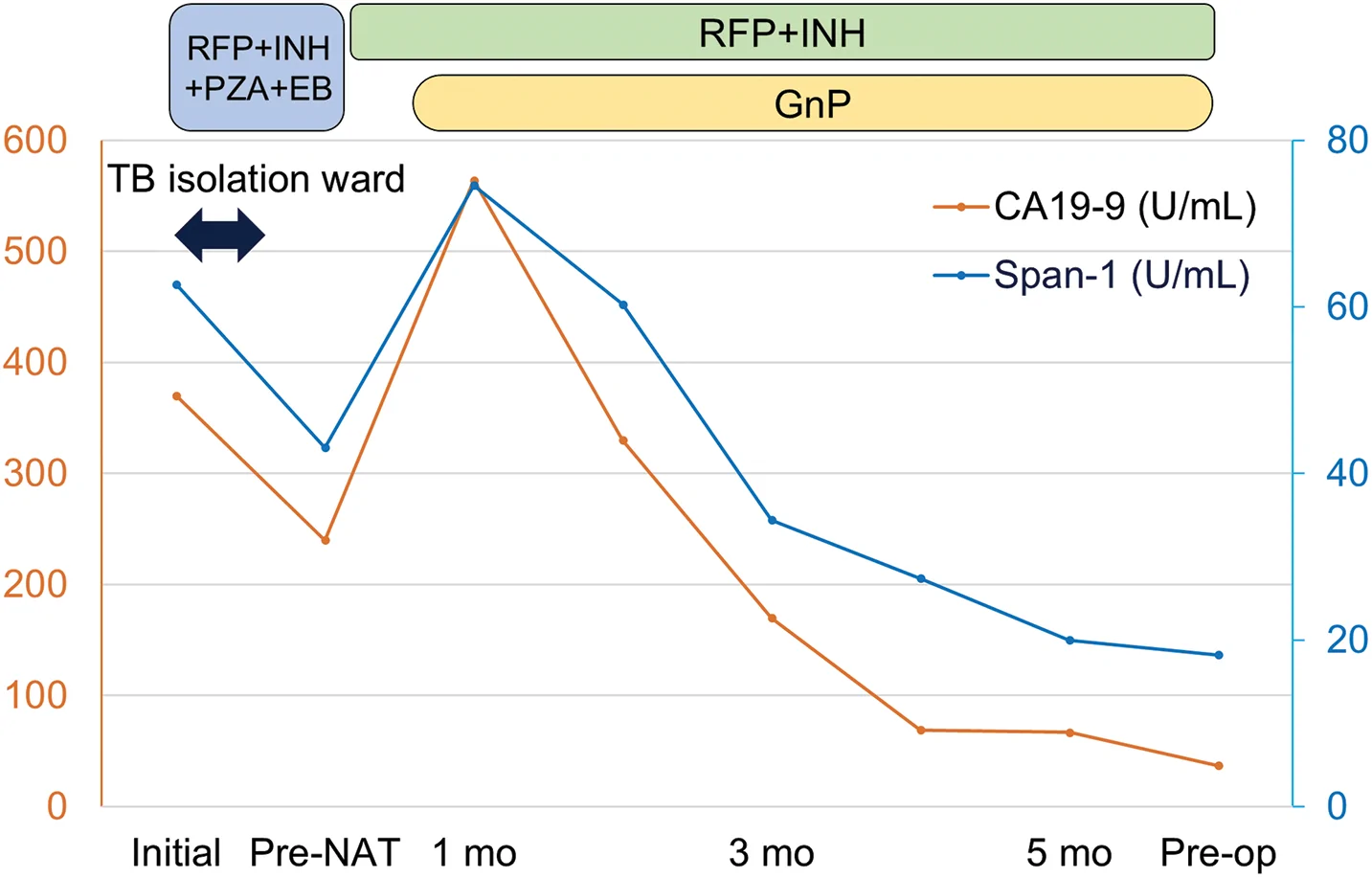
Treatment timeline and serum tumor marker kinetics. Following the initial 4-drug anti-TB therapy and sputum smear conversion, 6 cycles of neoadjuvant GnP were scheduled to coincide with the remaining course of maintenance 2-drug anti-TB therapy. CA19-9 and Span-1 levels decreased progressively during treatment. CA19-9, carbohydrate antigen 19-9; EB, ethambutol; GnP, gemcitabine plus nab-paclitaxel; INH, isoniazid; PZA, pyrazinamide; RFP, rifampicin; Span-1, s-pancreas antigen-1; TB, tuberculosis

GnP was completed as planned during maintenance anti-TB therapy. Mild neutropenia occurred during treatment but did not require treatment interruption. Although a mild drug eruption related to anti-TB therapy occurred, it resolved with symptomatic treatment, and the planned anti-TB therapy was completed. No clinical or radiological evidence of TB reactivation was observed. After completion of the planned GnP regimen, the CA19-9 and Span-1 levels normalized to 37 and 20 U/mL, respectively (**Fig. 3**). CT demonstrated tumor shrinkage from 21 to 14 mm with no change in portal vein abutment (**Fig. 2C** and **2D**). These findings met the criteria for a partial response according to the RECIST version 1.1.^8)^

The patient subsequently underwent subtotal stomach-preserving pancreatoduodenectomy with portal vein resection and reconstruction (**Fig. 4A** and **4B**). Severe intra-abdominal adhesions, thought to be related to previous tuberculous peritonitis, made the operation technically demanding. Lymph node dissection around the superior mesenteric artery was performed using a mesenteric approach.^9)^ Although the tumor had been classified as anatomically resectable preoperatively, dissection of the tumor from the portal vein proved difficult intraoperatively, suggesting portal vein invasion. Therefore, portal vein resection and reconstruction were performed. Digestive tract reconstruction was completed using a modified Child method. The operative time was 642 min, blood loss was 1710 mL, and 6 units of packed red blood cells were transfused. Histopathological examination revealed moderately differentiated tubular adenocarcinoma with invasion into the anterior and posterior peripancreatic tissues, common bile duct, and portal vein (**Fig. 4C** and **4D**). A histological therapeutic response was observed and graded as Grade 1b according to the Japan Pancreas Society criteria, corresponding to a minor pathological response.^10)^ An R0 resection was achieved, and 1 of 41 regional lymph nodes was positive for metastasis. The final pathological stage was yfT3N1aM0, Stage IIB according to the Japanese classification,^10)^ corresponding to ypT1N1M0, Stage IIB according to the UICC TNM Classification.^11)^

**Fig. 4:**
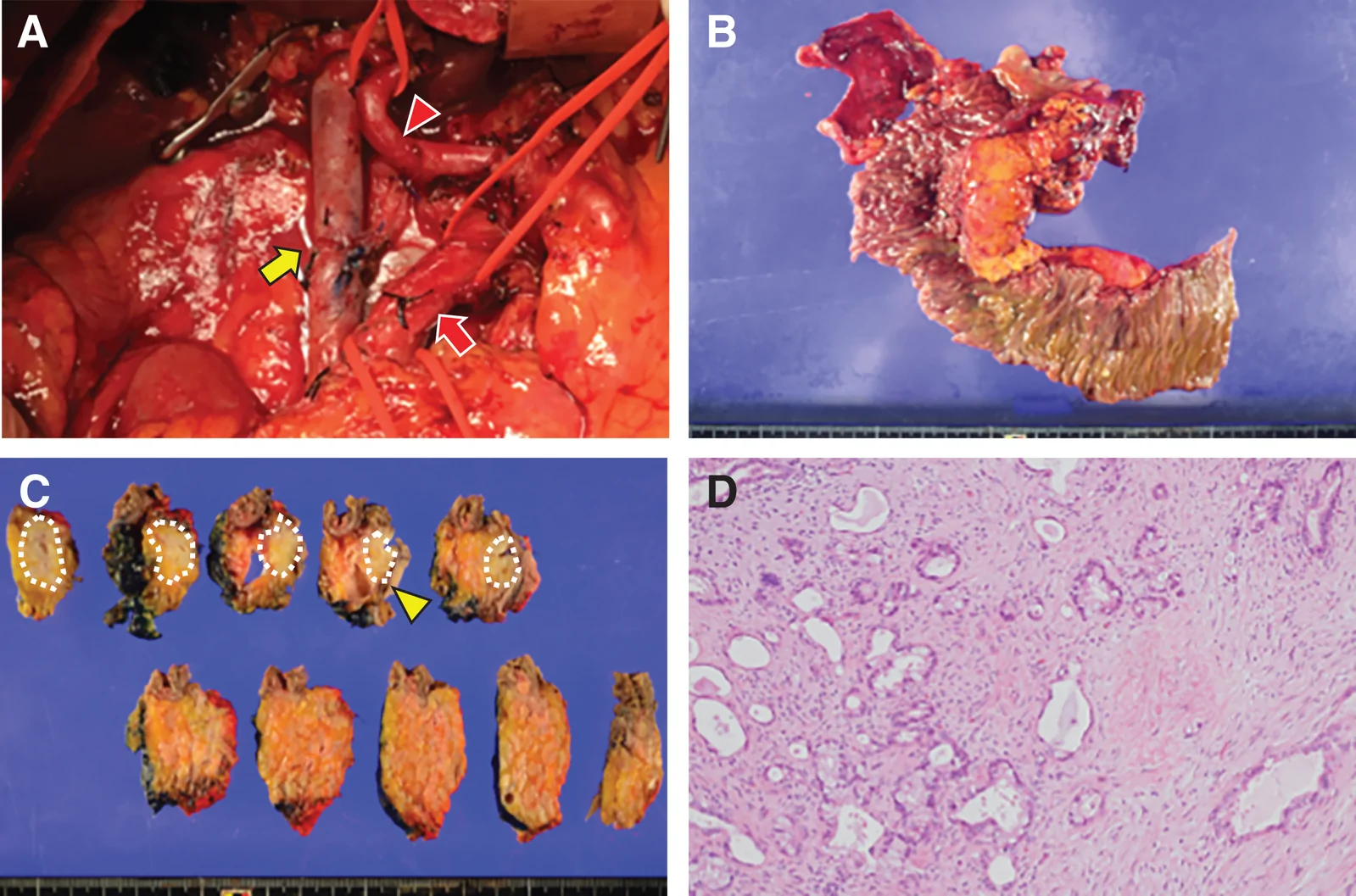
Surgical and pathological findings. (**A**) Subtotal stomach-preserving pancreatoduodenectomy with portal vein resection and reconstruction was performed. The reconstructed portal vein (yellow arrow), common hepatic artery (red arrowhead), and superior mesenteric artery (red arrow) are shown. (**B**) The resected sample is shown. (**C**) Histopathological mapping demonstrating a residual tumor (white dashed line) with portal vein invasion (yellow arrowhead). (**D**) Histopathological examination revealed irregular glandular structures with atypical, enlarged nuclei, consistent with moderately differentiated tubular adenocarcinoma. The histological therapeutic effect was graded as Grade 1b according to the Japan Pancreas Society criteria (hematoxylin and eosin staining, ×100).

The postoperative course was uneventful, and the patient was discharged on POD 21. Adjuvant S-1 therapy was administered for 6 months in accordance with the Japanese guidelines.^3)^ An isolated local recurrence was detected 16 months after surgery. Given the relatively long recurrence-free interval, GnP was reintroduced as rechallenge chemotherapy. At the latest follow-up, 24 months after surgery and 33 months after the initiation of anti-TB therapy, the patient remained alive with stable disease under ongoing chemotherapy, without evidence of TB reactivation.

## DISCUSSION

This case illustrates the clinical difficulty of managing PDAC when active pulmonary TB is diagnosed concurrently. In such situations, infection control, systemic chemotherapy, and major pancreatic surgery must be carefully coordinated to avoid losing the opportunity for curative-intent treatment. In this patient, sputum conversion and the confirmation of drug-susceptible TB were achieved after initial anti-TB therapy. A 6-month course of neoadjuvant GnP was subsequently completed according to the patient’s schedule during maintenance anti-TB therapy without TB reactivation, enabling pancreatoduodenectomy followed by adjuvant S-1 chemotherapy. Although a local recurrence developed, he remained alive with stable disease under subsequent systemic therapy 33 months after treatment initiation.

Active TB is increasingly recognized as a clinically relevant complication in patients with malignancy. Previous studies have shown that the risk of active TB is elevated after cancer diagnosis, particularly within the first several months, and that this risk varies according to cancer type.^2,12,13)^ Although pancreatic cancer accounts for a relatively small proportion of TB-associated malignancies, it has been reported as one of the high-risk solid tumors associated with the highest risk of active TB. This association may be explained by pancreatic cancer-related malnutrition, diabetes mellitus, impaired cell-mediated immunity, and the immunosuppressive effects of systemic therapy.^14)^ Despite this increased risk, reports describing the coexistence of pancreatic cancer and TB remain exceedingly limited. Although pancreatic TB has been well recognized as a diagnostic mimic of pancreatic cancer,^15)^ only a small number of reports have described pancreatic cancer coexisting with pancreatic TB or metastatic and palliative-stage pancreatic cancer complicated by pulmonary TB.^16,17)^ To our knowledge, this is the first reported case of PDAC with active pulmonary TB requiring concurrent treatment for both conditions before curative-intent resection. In the present case, active pulmonary TB was not simply an infectious comorbidity but a host-related factor that fundamentally influenced treatment planning, necessitating careful balancing of infection control and oncologic priorities.

Management of patients with concomitant active TB and malignancy requires balancing 2 competing risks. Cytotoxic chemotherapy before adequate infection control may exacerbate or disseminate TB, whereas excessive delay in cancer treatment may compromise the opportunity for curative-intent treatment.^18)^ This dilemma is particularly relevant in PDAC because of its aggressive clinical course and poor prognosis.^3)^ Nair et al.^18)^ reported that active TB delayed or modified cancer treatment in nearly half of affected patients and resulted in the loss of curative-intent treatment in a subset of cases. Conversely, several small retrospective studies, predominantly involving patients with lung cancer and other solid malignancies, have suggested that concurrent anticancer chemotherapy and anti-TB therapy can be feasible in carefully selected patients under close monitoring.^4,19,20)^ Consistent with the approach reported by Hirashima et al.,^4)^ we prioritized infection control before introducing neoadjuvant chemotherapy. Chemotherapy was not administered during active bacterial shedding and was initiated only after sputum conversion, confirmation of drug-susceptible TB by MGIT-based testing, and assessment of early tolerability to anti-TB therapy. With the initiation of chemotherapy, the potential impact of intermittent dexamethasone used for antiemetic prophylaxis on infection control was also discussed with the pulmonology team. Given that the risk associated with brief intermittent corticosteroid exposure remains uncertain, in contrast to the established risk with prolonged systemic corticosteroid therapy,^21)^ its use during effective anti-TB therapy was considered acceptable with careful monitoring. Importantly, these decisions were made through multidisciplinary discussion and shared decision-making with the patient because evidence supporting concurrent anti-TB therapy and anticancer treatment is limited to small retrospective studies in other malignancies, and no comparable experience has been reported in PDAC. Although tumor progression was observed during the initial phase of anti-TB therapy, neoadjuvant chemotherapy was promptly introduced after infectivity had resolved and drug-susceptible TB had been confirmed. As a result, both infection control and oncologic control were successfully maintained, leading to a partial radiological response, curative-intent resection, and subsequent completion of adjuvant S-1 therapy.

Concurrent anticancer chemotherapy and anti-TB therapy require careful attention to both treatment-related toxicity and drug‒drug interactions. Anti-TB therapy itself may cause adverse events, including hepatotoxicity associated with INH or RFP and optic neuritis associated with EB, which may necessitate treatment modification or discontinuation.^22)^ Therefore, assessment of early tolerability is important before introducing cytotoxic chemotherapy.^4)^ In the present case, only mild drug eruption occurred during anti-TB therapy and was successfully managed with symptomatic treatment, allowing completion of the planned anti-TB regimen. Drug‒drug interactions are another important consideration. RFP is a potent inducer of CYP enzymes and may reduce exposure to several anticancer agents through accelerated drug metabolism.^23)^ Nab-paclitaxel is an albumin-bound formulation of paclitaxel, which is metabolized primarily by CYP2C8 and CYP3A4, and concomitant treatment with RFP may theoretically decrease its systemic exposure. This potential interaction was recognized when GnP was selected, and treatment was initiated with careful monitoring of its antitumor efficacy. Nevertheless, marked decreases in serum tumor marker levels were observed during treatment, demonstrating clinical antitumor activity despite the potential interaction and supporting the continuation of GnP therapy. Ultimately, a partial radiological response and a Grade 1b histological response were achieved despite the concurrent administration of RFP-containing anti-TB therapy. Although the pharmacokinetic safety or efficacy of concurrent GnP and anti-TB therapy cannot be established from a single case, our experience suggests that this combination may be feasible under careful monitoring.

Although the tumor was anatomically resectable, active pulmonary TB represented a clinically important host-related factor that substantially increased the complexity of treatment planning. The patient did not strictly fulfill the formal IAP consensus criteria for BR-C because his performance status was 1; nevertheless, active TB had the potential to compromise resectability if infection control failed, performance status deteriorated, or oncologic treatment was excessively delayed. We therefore adopted a treatment strategy analogous to the BR-C concept, regarding the clinical situation as closer to borderline, high-risk disease than to conventional resectable PDAC because the prolonged period required for anti-TB therapy could jeopardize oncologic control and ultimately resectability.^5)^ In this framework, immediate surgery was not considered optimal; instead, we used the preoperative period to confirm control of TB, assess the patient’s tolerance to ongoing anti-TB therapy and chemotherapy, and maintain oncologic control with neoadjuvant treatment before proceeding to resection. This approach allowed careful patient selection and helped ensure that curative-intent surgery was undertaken only after both infectious and oncologic conditions were deemed acceptable. This strategy is also consistent with the concept proposed by Bates et al.,^24)^ in which the interval before cancer surgery may be repurposed “wait time” to “preparation time.” In this patient, gemcitabine plus S-1 was also considered as neoadjuvant regimen because it is an established treatment in Japan for patients with PDAC undergoing curative-intent treatment.^25)^ However, the prolonged preoperative period required for anti-TB therapy created a particular need for sustained oncologic control over approximately 6 months. Despite the aforementioned potential interaction between RFP and nab-paclitaxel, GnP was selected as an established intensive systemic regimen for unresectable or recurrent PDAC because of its efficacy and favorable tolerability, including in older patients,^7)^ together with emerging evidence supporting favorable outcomes in Japanese patients.^26)^ The successful clinical course observed in the present case suggests that, in carefully selected patients, the interval required for TB treatment need not be viewed as a delay to surgery but may instead serve as a valuable preparation time for curative-intent resection.

Several limitations should be acknowledged. As a single-case experience, the treatment strategy described here should not be generalized without careful consideration of TB infectivity, drug susceptibility, patient condition, tumor biology, and available infection control resources. In addition, the safety and efficacy of concurrent GnP and RFP-based anti-TB therapy remain uncertain because potential pharmacokinetic interactions could not be directly evaluated in this patient and clinical experience remains extremely limited. Importantly, the observed clinical response cannot exclude a clinically relevant reduction in nab-paclitaxel exposure. Further accumulation of similar cases is needed to establish optimal treatment strategies for patients requiring the concurrent management of active TB and PDAC.

## CONCLUSIONS

This case demonstrates that the concurrent management of active pulmonary TB and PDAC can be feasible when infection control and oncologic treatment are appropriately balanced. Until more robust evidence becomes available, multidisciplinary decision-making remains essential in these challenging clinical situations.

## DECLARATIONS

### Use of artificial intelligence tools

ChatGPT (OpenAI, San Francisco, CA, USA) was used to assist with English-language editing and manuscript refinement after the manuscript was drafted. No AI tool was used to generate clinical data, analyze data, or modify images. The authors carefully reviewed and edited all the AI-generated suggestions and take full responsibility for the final content of the manuscript.

### Availability of data and materials

All the data generated or analyzed during this study are included in the published article.
